# Supplementary material for: Comprehensive Annotation and Functional Exploration of MicroRNAs in Lettuce
Source: Front Plant Sci. 2021 Dec 24;12:781836. doi: 10.3389/fpls.2021.781836 (PMC8739914; doi:10.3389/fpls.2021.781836)
Supplement: Supplementary Data 1 — Results of categories 0–2 from PARE-Seq analysis (including three subfiles:1_1, 1_2, 1_3). [file Data_Sheet_10.ZIP › GSM2230755.plot/Lsa-miR2111_Lsat_1_v5_gn_2_4860.1_11831_TPlot.pdf]

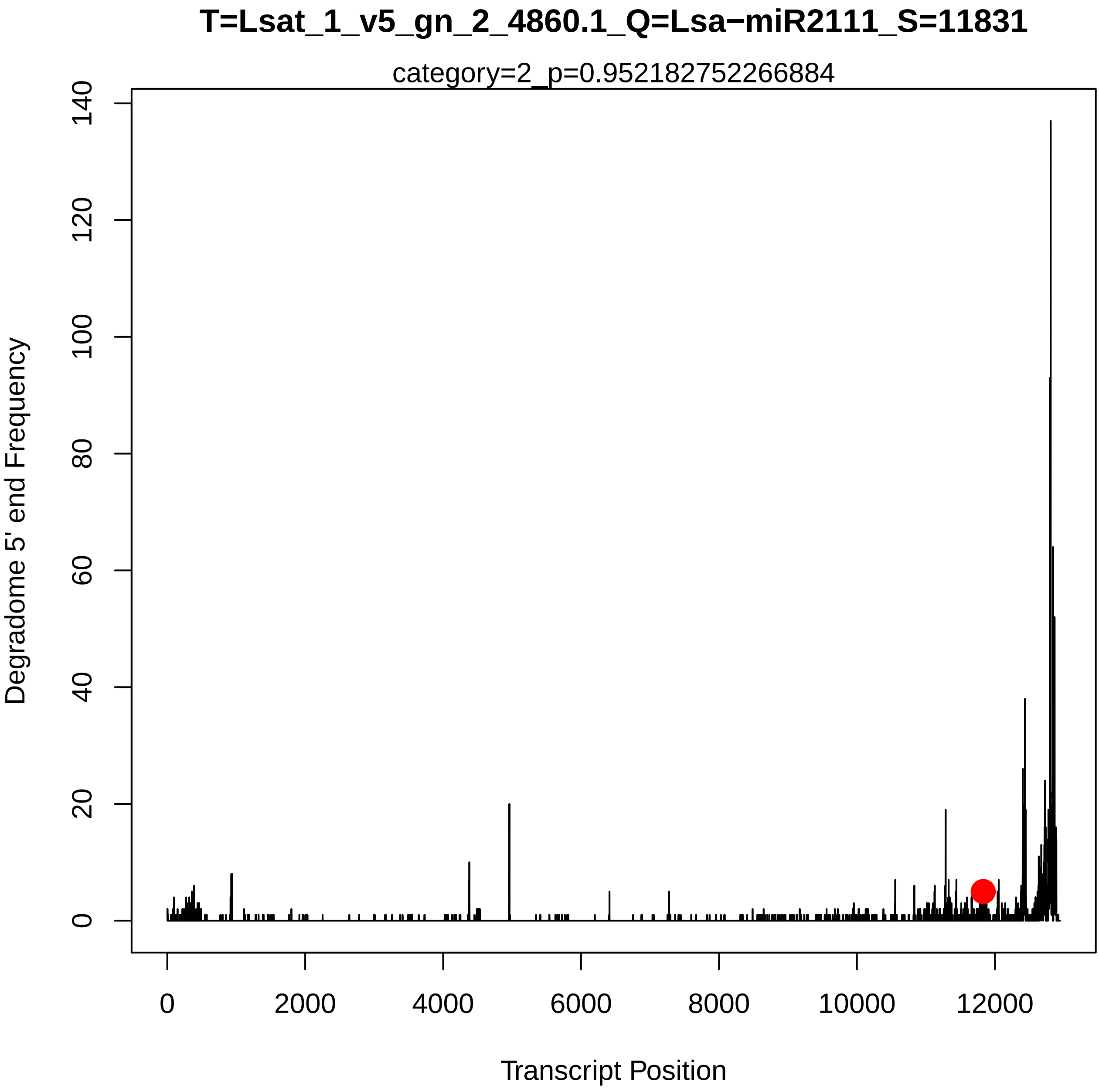
T=Lsat_1_v5_gn_2_4860.1_Q=Lsa−miR2111_S=11831
category=2_p=0.952182752266884
Degradome 5' end Frequency
0
20
40
60
80
100
120
140
0
2000
4000
6000
8000
10000
12000
Transcript Position
